# Popularity and Entropy in Friendship and Enmity Networks in Classrooms

**DOI:** 10.3390/e25070971

**Published:** 2023-06-23

**Authors:** Diego B. Sánchez-Espinosa, Eric Hernández-Ramírez, Marcelo del Castillo-Mussot

**Affiliations:** 1 Facultad de Ciencias, Universidad Nacional Autónoma de México, Ciudad de México 04510, Mexico; diegobalam@ciencias.unam.mx; 2 Instituto de Investigaciones Económicas, Universidad Nacional Autónoma de México, Ciudad de México 04510, Mexico; erichr@comunidad.unam.mx; 3 Instituto de Física, Universidad Nacional Autónoma de México, Ciudad de México 04510, Mexico

**Keywords:** popularity, friendship networks, enmity networks, weighted networks, local entropy

## Abstract

Looking for regular statistical trends of relations in schools, we constructed 42 independent weighted directed networks of simultaneous friendship and animosity from surveys we made in the Mexico City Metropolitan area in classrooms with students of different ages and levels by asking them to nominate and order five friends and five foes. However, the data show that older students nominated fewer than the five required five foes. Although each classroom was independent of the others, we found several general trends involving students of different ages and grade levels. In all classrooms, friendship entropy was found to be higher than enmity entropy, indicating that fewer students received enmity links than received friendship nominations. Popular agents exhibited more reciprocal nominations among themselves than less popular agents, and opposite-sex friendships increased with age.

## 1. Introduction

Networks have emerged as powerful tools for studying structures across various disciplines, including economics, physics, biology, and sociology. In the field of social sciences, networks have played a significant role, particularly in examining social interactions, a practice that dates back to the 1930s. Social networks serve as representations of individuals, with nodes symbolizing people and edges denoting social interactions such as friendship or enmity. Among these interactions, the study of friendship holds primary importance, as it is correlated with mental health and emotional and social development, and additionally serves as an indicator of success. However, defining the concept of friendship is a complex task that evolves over time. For children, friends are playmates, while for adolescents, they form a group of individuals with whom they engage in shared activities. In adulthood, friends are those with whom individuals have frequent interactions. Social relations play a crucial role, as they form the basis for the success of our society and species. Additionally, they have a significant impact on physical and mental wellbeing. Various studies have demonstrated that friendship is closely linked to social and emotional development [1,2,3,4,5].

When studying friendship, it is essential to observe the variables that determine or foster these relationships between individuals. Generally, friendship tends to occur between individuals who share activities, physical characteristics, and personality traits. This tendency to associate with similar individuals is known as homophily. Research suggests that sharing personality traits with an individual has a positive relationship with the likelihood of forming a friendship [6,7,8]. Moreover, the sharing of genotypes has been found to have a positive impact as well [9].

In the exploration of friendship relationships, researchers have commonly employed questionnaires to study student friendship networks [10,11,12]. These questionnaires gather information from each individual in a classroom by asking them to identify their friends. This process allows for the establishment of a popularity ranking within the classroom. This analysis has revealed the existence of two types of friendship structures, namely, reciprocal and non-reciprocal networks. These networks follow distinct formation dynamics, and have been associated with social status [11].

On the other hand, less attention has been paid to studying enmity or dislike relationships, despite their presence in issues such as ethnic segregation in school environments [13] and bullying [14]. A new type of network called signed graphs, which consist of both positive (friendship) and negative (enmity) relationships, has gained attention in contexts such as workplace gossip, voting patterns, international alliances, conflicts [15], and social media [16,17]. It is important to note that friendship and enmity do not affect only emotional aspects; they may have implications for physical health as well. For instance, obesity has been found to be negatively related to friendship and positively related to enmity [18]. Friendship has been shown to have a positive impact on academic and professional performance, contributing to academic development [19].

Researchers have made significant efforts to understand the patterns and dynamics of both friendship and enmity structures as a whole. In a school environment, networks of friendship and enmity relationships have been analyzed in elementary schools in Mexico, revealing similarities in the networks across different schools. It has been determined that friendship and enmity networks follow distinct degree distributions [20]. Based on this evidence, models have been proposed to determine the importance of homophily, transitivity, and the effects of preferential attachment in the formation of friendship networks [21]. In a different approach, a previous article examined groups that are internally friendly and mutually hostile in school classes in Mexico City, modeling group formation as an evolution towards Heider balance and identifying stronger gender segregation in younger classes [22]. Now, using the same dataset, the objective of this study is to find trends or patterns in those independent classrooms.

Using surveys, it is possible to analyze the level of homogeneity in friendship and enmity relationships within classrooms by employing the concept of local entropy in each classroom. First, each student is asked to nominate five friends and five foes in his/her classroom in order of intensity to generate an adjacency matrix with two types of links, in turn generating a weighted directed network with two types of “arrows”. The social entropy that calculated here is richer than if students are only been asked to name classmates with three types of interaction (friendship, enmity, and neutrality). As a simple analogy, a die tossed in the air has six possible outcomes, providing more information and entropy than a coin, which has only two outcomes.

Researchers have looked for variables in social systems that can be susceptible to analysis using the concept of entropy [23,24,25,26]. Different entropy measures are focused on network centrality measures, which have been used in the past in many social systems [27,28,29]. For analysis of the diversity of link weights in a network, researchers have proposed using the Shannon entropy to quantify topological diversity [30]. For this type of measure, high entropy means greater diversity while low entropy means little diversity. The more homogeneous situation in a network corresponds to having all weights equal (lowest entropy), and in extreme cases one single connection accumulates a disproportionate weight at the expense of all others [31,32].

Our research question here is to determine whether the social properties that are found based on the applied survey are more or less independent of variables such as the number of students in each classroom and their distribution by age and genres. To compare statistical properties in samples, there are several size effects: statistical effects, and in our case those due to social behavior, which can be further subdivided into the number of people and their composition by gender. Because students in the survey only mentioned peers of the same group as friends or enemies, each class approximates a small *closed universe* that is independent of the other groups. Therefore, an interesting line of research could be to analyze how behavior and interpersonal relationships depend on the number of people and the fraction of men and women. Studies such as the one we present here focusing specifically on this question would be useful in this regard.

The rest of this paper is organized as follows. In Section 2, we provide a detailed description of the datasets and methods used for analysis. In Section 3, we present and discuss the results. Finally, in Section 4, we present our conclusions.

## 2. Materials and Methods

**Figure 1:**
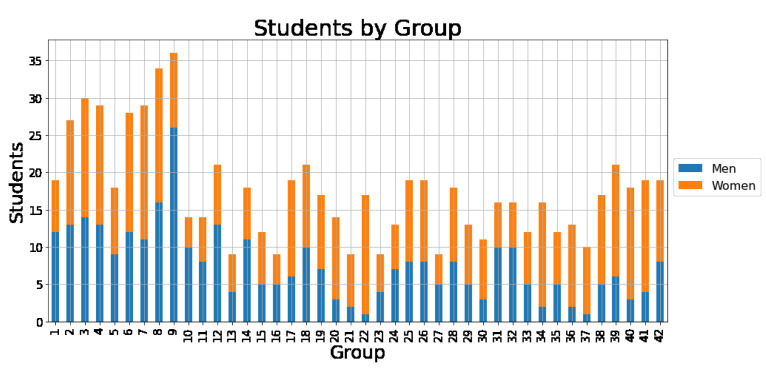
This bar plot displays the total number of students and teachers in each classroom, categorized by genre and ordered from the lowest average age starting from the left.

**Figure 2:**
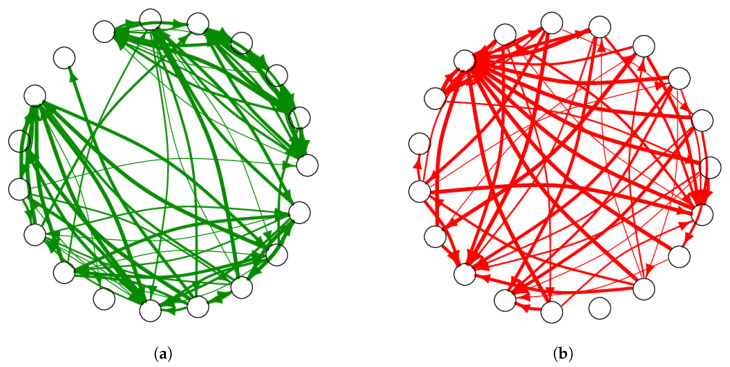
Example networks with only friendship and only enmity shown separately for the same classroom. The thickness corresponds to the intensity of the relationship, with thinner lines indicating intensity 1 and thicker lines indicating intensity 5. (**a**) Subnetwork with only friendship ties; (**b**) Subnetwork with only enmity ties.

The study was conducted in 42 independent classrooms, 38 of which were classrooms ranging from basic to university level of education and four of which consisted of teachers. Figure 1 shows the total number of students and teachers in each classroom categorized by genre. Written consent was obtained from the guardians of the children who participated in the study. The set of classrooms was randomly chosen; for convenience, most were located in schools near the university where we work. Furthermore, all data were analyzed in an anonymous manner upon arrival to the researchers. The average age of each group ranged from 9.88 to 39.58 years old. Figure 1 shows the composition of each classroom in terms of the number of students and gender. In each classroom, a questionnaire was applied to the students. Each participant was asked to name their five best friends and assign them an integer between 1 and 5, where 1 is the lowest level of friendship intensity and 5 is the highest. Similarly, they were asked about their five enemies or foes using the same degrees of intensity, which we considered negative relationships, i.e., −1 to −5. With the information from this survey, we constructed a directed and weighted network for each of the 38 classrooms and the four groups of teachers, then assigned each of the links an intensity in a range of integers between −5 to 5, with zero indicating no relationship, [−5,−1] indicating enmity, and [1,5] indicating friendship. In this sense, if the information was complete, we obtained simultaneous directed networks with negative (enmity) and positive (friendship) values, as shown in Figure 2.

Then, for each classroom, we defined a signed weighted network by means of the adjacency matrix W:

(1) $$w_{ij}=\left\{\begin{array}{cc} \left[-5,-1\right] & \;If\;there\;are\;enmity\;relationship\;from\;i\;to\;j\\ \left[1,5\right] & \;If\;there\;are\;friedship\;relationship\;from\;i\;to\;j\\ 0 & \;in\;other\;case. \end{array}\right.$$

where $w_{ij}$ is an integer in [−5,5].

**Figure 3:**
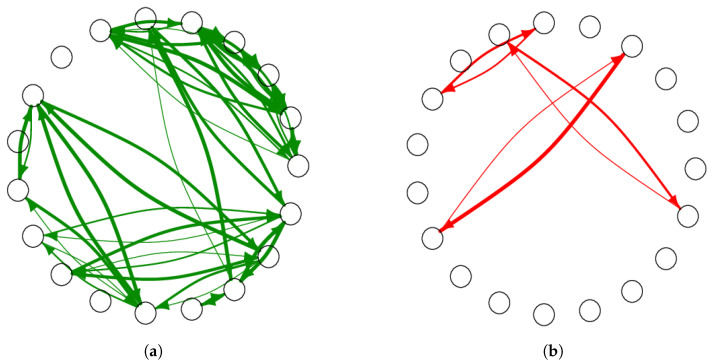
Same as Figure 2, except showing subnetworks with only reciprocal links. The thickness corresponds to the intensity of the relationship, with thinner lines indicating intensity 1 and thicker lines indicating intensity 5. (**a**) Subnetwork with reciprocal friendship ties; (**b**) Subnetwork with reciprocal enmity ties.

From the adjacency matrix W, we constructed four subnetworks (Figure 2 and Figure 3). Here, friendship networks have only positive ties and enmity network have only negative ones, while reciprocal networks only include links in both directions, i.e., both friendship (Figure 3a) and enmity (Figure 3b) ties.

### 2.1. Popularity and Reciprocity

In order to classify the members of each classroom into different levels of popularity, we utilized the number of positive nominations received by each student as a popularity measure. Specifically, we calculated the in-degree $k^{in}$ within the positive subnetwork for each student. Subsequently, we arranged them in descending order based on their $k^{in}$, thereby obtaining a comprehensive ranking of the members within each classroom. Based on this systematic arrangement, students were classified into three distinct categories, as follows: popular, average, and unpopular. In this case, the top 25 percent of students with the highest number of received friendships were designated as popular, the lowest 25 percent were categorized as unpopular, and the remaining students were classified as average. After the members of a classroom were classified into one of the three mentioned categories, it was possible to determine the number of friendly relationships each student had within each level of popularity. This procedure was carried out for the various subnetworks obtained based on gender, relationship, and age.

To analyze the percentage of reciprocity in friendships and hostilities as well as relationships between individuals of the opposite gender, we defined the ratio

(2) $$r_{i}=\frac{k_{reciprocal}^{in}}{k_{complete}^{out}},$$

where $k^{in}$ represents the degree of student *i* within the reciprocal network and $k^{out}$ denotes the number of edges departing from student *i* in the overall directional network. When $r_{i}$ equals one, all students nominated by student *i* reciprocally nominated student *i*. The percentage of reciprocity in the relationship network was analyzed based on popularity rating and gender.

For all cases, we used an analysis of variance model, where the independent variables were popularity classification, gender, and age. The dependent variables included relationships received (in-degree by popularity group), relationships given (out-degree by popularity group), and reciprocity in either friendship or enmity relationships. In addition, we examined reciprocal relationships with the opposite sex.

### 2.2. Structural Entropy

To assess the homogeneity of relationships in our weighted and directed networks, we started by determining the strength of the nodes. Recalling our assignment of signs (positive and negative) to the links using Equation 1, we can define the total strength of node *i* as the sum of its incoming strength ($T_{i}^{in}$) and outgoing strength ($T_{i}^{out}$) as follows:

(3) $$T_{i}=T_{i}^{in}+T_{i}^{out},$$

where

(4) $$T_{i}^{in}=\sum_{j\in v(i)}^{n}w_{ij}^{in},$$

(5) $$T_{i}^{out}=\sum_{j\in v(i)}^{n}w_{ij}^{out},$$

Obviously, the sum runs over the neighbors of node *i* (j∈v(i)). Using the strength, rather than just the degree, is an advantageous choice, as it captures the intensity of the connections, that is, the strength of friendship or enmity that a student has towards another. Recall that friendship links are represented here by positive numbers, while enmity links are represented by negative numbers; thus, a useful measure that enables us to determine the concentration of positive links that a node receives or emits is the local entropy of each node. This is defined as

(6) $$S_{i}^{in}=-\sum_{j\in v(i)}^{n}\frac{w_{ij}^{in}}{T_{i}^{in}}\log\frac{w_{ij}^{in}}{T_{i}^{in}}$$

(7) $$S_{i}^{out}=-\sum_{j\in v(i)}^{n}\frac{w_{ij}^{out}}{T_{i}^{out}}\log\frac{w_{ij}^{out}}{T_{i}^{out}}.$$

Finally, the measure of local entropy for node *i* is provided by

(8) $$S_{i}=S_{i}^{in}+S_{i}^{out}.$$

This measure is close to 0 if the strength of node *i* is concentrated in a single incoming or outgoing link, and is close to 1 for homogeneous input and output weights. Each of the two classroom average entropies are the sum of the individual entropies divided by the number of members:

(9) $${\bar{S}}_{i}^{in}=\frac{\sum_{k}S_{k}^{in}}{m_{i}}$$

and

(10) $${\bar{S}}_{i}^{out}=\frac{\sum_{k}S_{k}^{out}}{m_{i}}$$

where $m_{i}$ is the number of agents in classroom *i*.

## 3. Results

### 3.1. Subnetworks

To focus on subgraphs with positive ties as a means of analyzing the average number of friendly nominations, we calculated the number of friendly nominations received (in-degree) and the number of friendly nominations sent (out-degree) within each classroom for every subset in the popularity classification described in Section 2.1, namely, unpopular, average, and popular.

To determine the population mean of the different cases, we implemented an ANOVA (analysis of variance model) analysis, allowing us to obtain a mean for the different analyzed cases considering the different sizes of the classrooms. More information about the implementation of the ANOVA method can be found in [33]. Furthermore, most statistical software includes this type of statistical analysis [34]. The results are shown in Table 1, Table 2 and Table 3, and are discussed further below.

In Table 1, the Only Friendship block shows that on average an unpopular student received 1.27 friendship nominations, an average student 3.68, and a popular student 6.25 (in-degree per popularity classification). In the case of friendships sent (mean out-degree) for each popularity level, unpopular students nominated an average of 3.17 friendships, average students nominated an average of 3.99, and popular agents nominated an average of 4.17. Friendship with the opposite sex increased slightly with popularity.

Next, we focus on subgraphs with negative ties to analyze the average number of received enmity nominations (in-degree) and the number of sent enmity nominations (out-degree) for each subset in our popularity classification. The *Only Enmity* block of Table 1 reveals a decrease in received enmity with popularity. Specifically, unpopular students received an average of 4.51 enmity nominations, average students received 3.04 nominations, and popular students received 1.78 nominations. On the other hand, when assigning enmity (out-degree), the opposite effect is observed, with the average number of enmity nominations submitted increasing with popularity. Unpopular students had an average enmity nomination of 2.46, average students 3.12, and popular students 3.26. There is a significant difference between the mean for popular and unpopular students, with no significant difference between popular and average students. Regarding enmity towards the opposite sex, we observe a similar trend to overall enmity, with popular students exhibiting higher levels of enmity. However, when we consider the ratio of enmity towards the opposite sex compared to general enmity, we find that the percentages are very similar across all three popularity levels. Popular students allocate 47.85% of their enmity towards the opposite sex, average students 47.11%, and unpopular students 46.74%. Therefore, there is no significant difference in enmity towards the opposite sex compared to enmity towards members of the same sex within any of the popularity groups.

In the *Reciprocity* block, we find that popular students have more reciprocal nominations among themselves than less popular ones: unpopular students have a reciprocal percentage of 21%, average students 52%, and popular students 70%.

The *Popularity in Reciprocal Friendship* block in Table 1 displays the average number of reciprocal relationships among different popularity groups. The results reveal that there is little variation in the distribution of mutual friends based on popularity level. On average, unpopular students have 0.10 mutual friends who are also unpopular, accounting for 15.38% of their mutual friendships. They have 0.34 mutual friendships with average students, representing 52.30%, and 0.21 mutual friendships with popular students, representing 31.30% of their mutual friendships.

For average agents, the distribution of mutual friends yields 8.13% with unpopular students, 49.28% with average students, and 42.58% with popular students. Among popular students, the distribution of mutual friendships yields 5.78% with unpopular students, 55.10% with average students, and 39.11% with popular students. Generally, popular students have more mutual friendships across all levels of popularity.

Unpopular students tend to have more mutual friends who are also unpopular compared to average and popular students. However, the percentage of mutual friends is similar for all popularity groups; unpopular students have fewer popular friends compared to the other two groups.

We find that average reciprocity enmity is affected by popularity; popular students tend to assign more enmity and receive less. Their reciprocity percentage is 22%, while for average students it is 32% and for unpopular students it is 39%.

In principle, because every student in a classroom with N members was asked to nominate five friends and five foes, the total number of friendship and enmity links must be 5×N in both cases. Then, on average, every student would receive five nominations of each type. However, the data show that certain students nominated less than five people. For this reason, the average for both received and sent links is less than five. Table 2 shows very slight differences between males and females when analyzing the differences between friendship and enmity. On average, children experience greater friendship relationships with members of the same gender. Females nominated an average of 3.75 friends, of whom 1.24 were male, representing 33.06%. However, the percentage of reciprocity of friendship with the opposite gender is not different from the general percentage, as for females the overall percentage of reciprocity was 53% and the percentage of reciprocity with the opposite sex was 44%. In the case of males, the overall percentage was 51% and the percentage of reciprocity with the opposite sex was 49%. Table 3 shows the average results over classroom of various networks partitioned by age. The *Only friendship* block shows how positive ties are in three groups: the first from 9 to 15 years, the second from 15 to 20 years, and the last over 20 years of age. The average received friendship has a negative relationship with age, with the youngest group having an average of 4.51 friendship nominations, the 15–20 years old group having 3.76 m and the group over 20 years old having 3.48. In the last row of this block, it can be observed that friendship with the opposite sex increases with age; the younger group has 0.95 average friends of the opposite sex, the age 15–20 group has 1.38, and those over 20 have 1.65. If we analyze the number of opposite-sex friends as a percentage, we find that in the youngest group friendships with the opposite sex represent 21.34% of friendships, while for the age 15–20 group this is 36.70% percent and for the over 20 group it is 47.68%. Thus, opposite-sex friendships increase with age.

Next, we consider the network with negative links in the *Only Enmity* block of Table 3. Sent and received enmity is reduced with age; the average enmity received for the youngest group is 3.85 nominations, for the 15–20 group it is 2.94, and for the over 20 group it is 2.72. This shows that there are no significant statistical differences between the 15–20 and over-20 age groups, while there is a significant statistical difference for the 9–15 group. Enmity with the opposite sex likewise decreases with age. However, when we consider the percentage of total enmity assigned to the opposite sex, it is close to 50% for all age groups. The youngest group allocates 56.75% of their enmity nominations to the opposite sex, the 15–20 group 45.91%, and the over-20 group 43.47%. There is a significant difference in the means between these groups, except between the 15–20 group and the over-20 group.

Finally, the *Reciprocity* block in Table 3 shows that the level of average friendship and enmity reciprocity does not vary between age groups, while opposite-sex reciprocity increases with age. The reciprocity of enmity is lower in the youngest group of 9–15, with an average of 24 percent, while in the other two groups the average is very similar, with 35% for the 15–20 group and 34% for over-20 group. Thus, while the younger group receives and sends more enmity nominations, they have a lower percentage of reciprocity. When analyzing the number of people with whom enmity is reciprocal, on average individuals have one person with whom enmity is reciprocal. The difference in mean reciprocity is significant, while there is a significant difference in the mean of the youngest group compared to the means of the other two groups.

### 3.2. Entropy in Friendship and Enmity Networks

**Figure 4:**
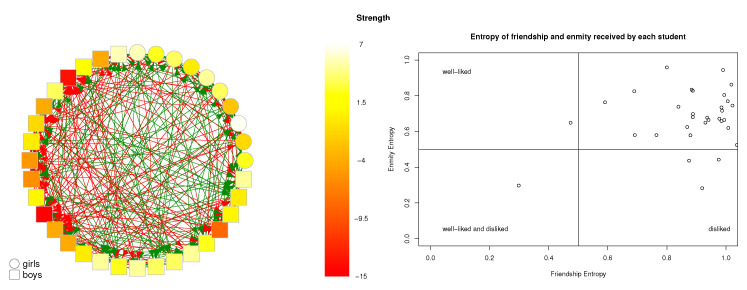
(**Left panel**): classroom networks with 36 nodes (students) indicated by gender, average age 13.74 years old. Node colors correspond to the total strength, with red indicating negative total strength (indicative of enmity) and white indicating positive total strength (indicative of friendship). (**Right panel**): corresponding scatterplot of received individual friendship and enmity entropy values. Only those members with nonzero receiving input links for both friendship and enmity are shown.

**Figure 5:**
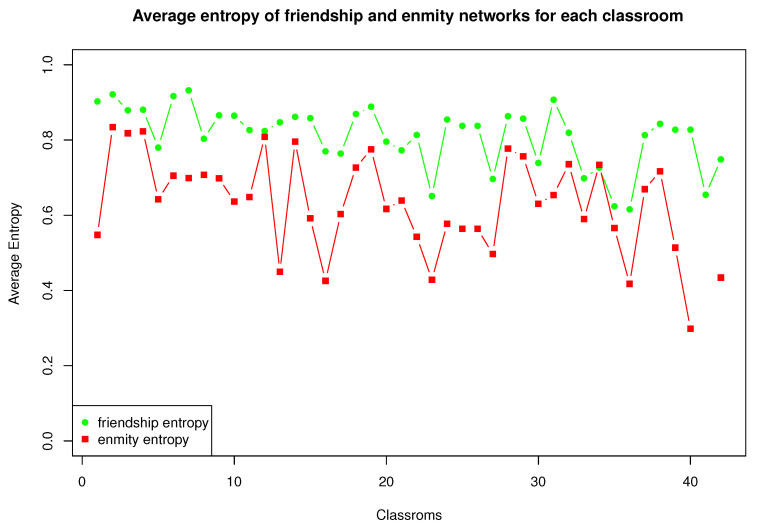
Average input entropy values for friendly relationships (green) and enmity relationships (red) of 42 classrooms networks, sorted in ascending order from left to right according to average age.

From Equations (6)–(8), we can determine the local entropy for each of the 38 classrooms and four groups of teachers. The right side of Figure 4 shows four quadrants, with most of the points concentrated in the upper right-hand corner, indicating that most of the individuals received friendship and enmity links from many others (i.e., high entropy). Only one student, located at the lower left-hand corner, received few friendship and enmity links (i.e., low entropy). Three students, situated at the lower right-hand corner, received relatively more friendship than enmity links. In this classroom, received friendship links are more homogeneous than the enmity links, as a large majority of the points in the upper right-hand quadrant lie below the diagonal. This same property is observed in all classrooms, as depicted in Figure 5.

## 4. Conclusions

We created 42 weighted directed networks of simultaneous friendship and animosity from surveys we made in the Mexico City Metropolitan area in 42 independent classrooms by asking students to nominate and order five friends and five foes. With the information from this survey, we constructed a directed weighted network for each of the 38 classrooms and four groups of teachers, where we assigned to each of the links an intensity in a range of integers between −5 to 5, with zero indicating no relationship, (−5, −1) enmity, and (1, 5) friendship. Thus, within each classroom the total number of friendship and enmity links must be 5×N in both cases; on average, every student should receive five nominations of each type. However, the data show that certain students, particularly in classrooms of older students, nominated fewer than five classmates. For this reason, the average of both received and sent links was less than five. It can be noted that in Table 3 this occurs more often for older students, who commented that they had very few foes because they got along very well with their classmates. It is possible that students may have doubted that the survey results were truly anonymous, or may have been were afraid that the survey results could be used for harmful purposes. In order to classify the members of each classroom into different levels of popularity, we utilized the number of positive nominations received by each student as a popularity measure.

Based on this systematic arrangement, each student was classified into distinct categories, namely, popular, average, and unpopular. The top 25 percent of students with the highest number of received friendships were designated as popular, the lowest 25 percent were categorized as unpopular, and the remaining students were classified as average. After classifying the members of a classroom into one of the three categories, we determined the number of friendly relationships each student had within each level of popularity. In the friendship subnetwork of popular students, students sent more enmity links within the same set of popular students, including opposite-sex students. Friendship was found to be more reciprocal among popular students. By gender, the number of friendships sent and received were both higher in males, especially at younger ages. Opposite-sex friendships increased and opposite-sex enmity decreased with age; however, practically neither friendship nor enmity reciprocity depended on age, while opposite-sex reciprocity increased with age.

In all classrooms, friendship entropy was found to be higher than enmity entropy, indicating that fewer students received more enmity links than received friendship nominations. Popular students exhibited more reciprocal nominations among themselves than less popular students, and opposite-sex friendships increased with age. Another important point to highlight is that in every classroom the average entropy values was higher for friendly relationships than for enmity relationships, as shown in Figure 5. As a detailed example, Figure 4 shows the received individual friendship and enmity entropy values in one classroom.

Our study has presented an array of empirical results obtained from personal surveys about friendship and enmity to build weighted directed networks including both friendship (positive) and enmity (negative). In summary, although each classroom was independent of the others, we found several interesting general statistical trends in the classrooms. However, this analysis is entirely descriptive; we do not discuss the mechanisms that may have generated the complex networks we analyzed. Clearly, it is desirable to perform similar studies as a function of time over shorter or longer periods (i.e., monthly, quarterly). In other words, the temporal evolution together with more personalized additional information can provide new elements for analysis. Obviously, the structure of communities within each classroom may influence the development of friendship and enmity relations. Overall, our study provides insights into the social dynamics of school groups, which could help educators and policymakers to design interventions to promote positive relationships among students.

Although the study was conducted in different schools in Mexico City, the finding that entropy is always lower in enmity networks than in friendship networks in each classroom (a robust behavior) indicates an interesting direction of research for other social groups.

When applying the same survey internally within each classroom, we obtained fairly similar results in all of them. It should be considered that there are many external interactions, however, as each classroom is not in fact a “closed universe”. In addition to digital social media interactions between people in different classrooms, there are personal interaction outside them, while entering or leaving the school grounds, in hallways, or during a recess. Furthermore, with regard to the different types of many-body social structures, there may be rivalries or alliances among members of different classrooms, or even with other schools, that could modify student preferences and links among them. It is necessary, of course, to carry out more in-depth analysis considering information about other features of students beyond age and gender, such as ethnic origin, economic or social status, reduced abilities, etc. How different might the results be between public and private schools, or by regions or countries? From the point of view of intertwined friendship and enmity webs, all these matters remain open research topics.

## Figures and Tables

**Table 1:** Average Over Classroom Partitioned Into Popular, Average, And Unpopular In Various Networks.

	Popularity		
	Unpopular	Average	Popular
	Mean	Mean	Mean
**Only Friendship**			
Received Friendship	1.27	3.68	6.25
Given Friendship	3.17	3.99	4.17
Opposite-sex friendship	1.17	1.33	1.43
**Only Enmity**			
Received Enmity	4.51	3.04	1.78
Given Enmity	2.46	3.12	3.26
Opposite-sex Enmity	1.15	1.47	1.59
**Reciprocity**			
Friendship	0.21	0.52	0.70
Enmity	0.39	0.32	0.22
Opposite-sex friendship	0.05	0.20	0.28
**Popularity in Reciprocal Friendship**			
Unpopular	0.10	0.17	0.17
Average	0.34	1.03	1.62
Popular	0.21	0.89	1.15
**Popularity in Reciprocal Enmity**			
Unpopular	0.22	0.25	0.18
Average	0.52	0.47	0.43
Popular	0.21	0.25	0.11

**Table 2:** Average Over Classroom Of Various Networks Partitioned By Gender.

	Gender	
	Female	Male
	Mean	Mean
**Only Friendship**		
Recieved Friendship	3.64	4.15
Given Friendship	3.75	4.00
Opposite-sex Friendship	1.24	1.44
**Only Enmity**		
Recieved Enmity	2.87	3.20
Given Enmity	2.94	3.12
Opposite-sex Enmity	1.30	1.59
**Reciprocity**		
Friendship	0.53	0.51
Opposite-sex Friendship	0.44	0.49
Enmity	0.29	0.29
**Popularity in Reciprocal Friendship**		
Impopular	0.16	0.15
Average	1.06	1.02
Popular	0.76	0.88
**Popularity in Reciprocal Enmity**		
Impopular	0.23	0.21
Average	0.50	0.44
Popular	0.21	0.19

**Table 3:** Average Over Classroom Of Various Networks Partitioned By Age.

	Range Age		
	9–15	15–20	20-
	M	M	M
**Only Friendship**			
Recived Friendship	4.51	3.76	3.48
Given Friendship	4.45	3.76	3.46
Opposite-sex Friendship	0.95	1.38	1.65
**Only Enmity**			
Recived Enmity	3.85	2.94	2.72
Given Enmity	4.0	2.94	2.76
Opposite-sex Enmity	2.27	1.35	1.20
**Reciprocity**			
Friendship	0.51	0.58	0.51
Enmity	0.24	0.35	0.34
Opposite-sex Friendship	0.33	0.55	0.49

## Data Availability

Data will be provided upon request to the corresponding author.
